# Beneficial effects of high dose taurine treatment in juvenile dystrophic mdx mice are offset by growth restriction

**DOI:** 10.1371/journal.pone.0187317

**Published:** 2017-11-02

**Authors:** Jessica R. Terrill, Gavin J. Pinniger, Keshav V. Nair, Miranda D. Grounds, Peter G. Arthur

**Affiliations:** 1 School of Molecular Sciences, the University of Western Australia, Perth, Western Australia, Australia; 2 School of Human Sciences, the University of Western Australia, Perth, Western Australia, Australia; Faculty of Biochemistry, Biophysics and Biotechnology, Jagiellonian University, POLAND

## Abstract

Duchenne Muscular Dystrophy (DMD) is a fatal muscle wasting disease manifested in young boys, for which there is no current cure. We have shown that the amino acid taurine is safe and effective at preventing dystropathology in the mdx mouse model for DMD. This study aimed to establish if treating growing mdx mice with a higher dose of taurine was more effective at improving strength and reducing inflammation and oxidative stress. Mice were treated with a dose of taurine estimated to be 16 g/kg/day, in drinking water from 1–6 weeks of age, after which *in vivo* and *ex vivo* muscle strength was assessed, as were measures of inflammation, oxidative stress and taurine metabolism. While the dose did decrease inflammation and protein oxidation in dystrophic muscles, there was no improvement in muscle strength (in contrast with benefits observed with the lower dose) and growth of the young mice was significantly restricted. We present novel data that a high taurine dose increases the cysteine content of both mdx liver and plasma, a possible result of down regulation of the taurine synthesis pathway in the liver (which functions to dispose of excess cysteine, which is toxic). These data caution that a high dose of taurine can have adverse effects and may be less efficacious than lower taurine doses. Therefore, monitoring of taurine dosage needs to be considered in future pre-clinical trials, in anticipation of using taurine as a clinical therapy for growing DMD boys (and other conditions).

## Introduction

Duchenne Muscular Dystrophy (DMD) is a lethal, X-chromosome linked muscle disease affecting about 1 in 3500–6000 boys worldwide (Reviewed in [1, 2]). DMD is caused by mutations in the dystrophin gene resulting in the loss of functional dystrophin protein in muscle, which leads to increased susceptibility of sarcolemmal damage and necrosis after muscle contraction [3, 4]. Consequently, dystrophic muscle undergoes repeated cycles of myofibre necrosis, associated with subsequent inflammation, fibrosis and regeneration: where this damage persists over many months and years in DMD boys it results in the replacement of muscle with fat and fibrotic tissue [5, 6]. The severe loss of muscle mass and function in DMD boys leads to premature death often due to respiratory or cardiac failure (reviewed in [1, 7]). While much effort has been dedicated to developing genetic or molecular therapies to replace the missing dystrophic protein [8, 9], there is no cure yet for DMD: the standard drug treatment using corticosteroids is limited in efficacy and associated with severe side effects [10, 11]. Consequently, there is considerable interest in developing safe and efficacious therapeutic drug or nutritional interventions that can prevent or slow the progression of DMD (reviewed in [12–14]).

The semi-essential amino acid taurine (2-aminoethanesulfonic acid) has been investigated as a pharmacological intervention, using the mdx mouse model of DMD [15–21]. In these studies, administration of taurine to mdx mice prevented myofibre necrosis, improved both *in vivo* and *ex vivo* muscle strength, increased fatigue resistance and decreased other indices of mdx pathology such as inflammation and oxidative stress. Recently we showed that an oral dose of approximately 4 g/kg/day (2% in drinking water, from 18 days until 6 weeks of age) had benefits on many indices of dystropathology (with improved *in vivo* and *ex vivo* muscle strength and decreased inflammation and oxidative stress) in mdx mice [19]. Research by De Luca and colleagues in 2003 established that treating male mdx mice aged 4 weeks (until 8 weeks) with a high dose of taurine (up to 400 mg per mouse per day, equivalent to about 16 g/kg/day calculated for a mouse weighing 25 g) was highly effective at reducing exercise induced muscle weakness [16].

We propose that one important mechanism of action of taurine in protecting mdx muscle from necrosis relates to effects on neutrophils (inflammatory cells that rapidly arrive at sites of damage). Specifically, taurine scavenges hypochlorous acid (HOCl), a highly reactive oxidant that is produced by the interaction of hydrogen peroxide and myeloperoxidase (MPO), a heme protein secreted by neutrophils [22]. HOCl can cause irreversible oxidative damage to proteins, as well as cause reversible protein thiol oxidation, which can dramatically modify protein function [23]. The scavenging of HOCl by taurine results in the formation of taurine chloramine, a molecule that itself exerts anti-inflammatory effects such as inhibiting the production of pro-inflammatory cytokines and nitric oxide (NO), and the inhibition of NF-κB activation by the oxidation of IκB-α [22, 24]. We have previously shown that muscle from the Golden Retriever Muscular Dystrophy (GRMD) dog model of DMD undergoes excessive neutrophil infiltration, leading to increased MPO content and HOCl production, which is associated with increased protein oxidation [25]. Likewise, we show that increased MPO content and protein thiol oxidation in mdx muscle is ameliorated in mice treated with a taurine dose of 4 g/kg/day (as discussed above) [19]. Therefore, the effects of a higher dose of taurine, estimated to be about 16 g/kg/day, on neutrophil and MPO content and protein thiol oxidation in mdx muscle were examined in the current study.

The aim of this study was to test if a high dose of taurine (16 g/kg/day) in young growing mdx mice was highly effective at reducing dystropathology, specifically for improving muscle strength and decreasing inflammation and protein thiol oxidation and to discern if these benefits were associated with decreased neutrophil and MPO content. Since we have shown previously that taurine metabolism (including synthesis of taurine in the liver from cysteine) is perturbed in mdx mice [26], we also assessed the effects of this high taurine dose on the content of taurine and cysteine in the liver, plasma and muscle of mdx mice, and examined taurine synthesis in the liver.

## Materials and methods

All reagents were obtained from Sigma-Aldrich unless otherwise specified.

### Taurine treatment

All animal experiments were conducted in strict accordance with the guidelines of the National Health and Medical Research Council Code of practice for the care and use of animals for scientific purposes (2004) and the Animal Welfare act of Western Australia (2002) and were approved by the Animal Ethics committee at the University of Western Australia.

All experiments were carried out on dystrophic mdx (C57Bl/10ScSnmdx/mdx) and non-dystrophic control C57 (C57Bl/10ScSn) mice (the parental strain for mdx). Mice were obtained from the Animal Resource Centre, Murdoch, Western Australia. Mice were maintained at the University of Western Australia on a 12-h light/dark cycle, under standard conditions, with free access to food and drinking water.

Mothers (and pups) were given either no treatment or taurine (delivered by 8% in the drinking water) from 7 days after birth, and taurine treatment continued after weaning (at 21 days) until they were sampled at 6 weeks. There were 9 mice in the C57 group, 10 in the untreated mdx group and 8 in the taurine treated group, however 8 mice of each group were randomly chosen for biochemical analysis. Several mice were removed from the *ex vivo* force measurements because the mice died under anaesthesia before the muscle could be removed. Both male and female pups were used in the study with equal numbers of both gender in each group. There were no significant effects of gender observed, except for liver (C57) and quadriceps (untreated mdx) weights (see S1 Table). Water ingestion and body weights were monitored twice weekly for all mdx treatment groups. There was no difference in water consumption of untreated and taurine treated mdx mice (averaging 3.2±0.5 and 4±2.3 ml per mouse per day respectively). The approximate consumption of taurine, based on water consumption, was about 16 g/kg/day.

### Grip strength

The grip strength of all mice was measured using a Chatillon Digital Force Gauge (DFE-002) and a triangle metal bar, as per the TREAT-NMD recommended standard protocol “Use of grip strength meter to assess limb strength of mdx mice– M.2.2_001 “http://www.treat-nmd.eu/downloads/file/sops/dmd/MDX/DMD_M.2.2.001.pdf”. In brief, the mouse was placed on the front of the triangle bar (attached to a force transducer) and pulled gently until released. Each mouse underwent 5 consecutive grip-strength trials; the grip strength value for each mouse was recorded as the average of the three trials with the highest force. Average grip strength was presented as total force (g) and also normalized for body weight [Force (g)/BW (g)]. Grip strength measurements were performed on three separate occasions during the final week of treatment to accustom mice, however only data from the final testing session was used for analysis.

### Tissue collection

Mice (at 6 weeks of age) were anaesthetized via an intraperitoneal (IP) injection of sodium pentobarbitone (40 mg/kg body weight). Anaesthetized mice were placed on a heated plate at 37°C to maintain core body temperature. Following dissection of the EDL muscle (for physiological measurements of contractile function), blood was removed via cardiac puncture and other tissues, including the quadriceps muscle and liver, were removed and snap frozen for further analysis.

*Ex vivo* muscle function testing was performed as per [27]. In brief, the EDL tendons were tied with non-absorbable black braided surgical silk (Dysilk, Dynek Pty Ltd) and mounted in an *in vitro* muscle test system (805A In Vitro Force Transducer System, Aurora Scientific Inc.) filled with mammalian ringer solution (containing (in mM) NaCl (121); KCl (5.4); MgSO_4_.7H_2_O (1.2); NaHCO_3_ (25); HEPES (5); glucose (11.5) and CaCl_2_ (2.5)), bubbled with Carbogen and maintained at 25°C. The muscle was stimulated with 0.2 ms square wave pulses (701B High Power Bi-phase Current Stimulator, Aurora Scientific Inc.) via parallel electrodes situated on both sides of the suspended muscle.

### *Ex vivo* measurement of force

At the start of each experiment the muscle was set to the optimal length (L_o_) by manually adjusting the lever arm and recording the twitch force response. The length at which the maximum twitch force was recorded was taken as L_o_. All subsequent measurements of contractile function were performed at L_o_. The optimal fibre length (L_f_) in the EDL was calculated from a pre-defined fibre-length to muscle-length ratio of 0.44 [28]. The force frequency relationship was evaluated by recording the force responses at stimulation frequencies of 5, 10, 20, 30, 50, 80, 100, 120 and 150 Hz. Each stimulus was separated by a two minute interval to avoid inducing muscle fatigue. The stimulation that produced the greatest force throughout the experiment was recorded as the maximum tetanic force.

Force recordings were normalized relative to muscle cross sectional area (CSA) and presented as specific force (N/cm^2^). CSA was estimated by dividing the wet muscle mass (g) by L_f_ and the density of mammalian skeletal muscle (1.056 mg / mm^3^).

### Inflammation

Neutrophil presence in quadriceps muscles was measured using immunoblotting, as described below. MPO catalyses the production of hypochlorous acid from hydrogen peroxide and chloride [29] and hypochlorous acid reacts with 2-[6-(4-aminophenoxy)-3-oxo-3H-xanthen-9-yl]benzoic acid (APF) to form the highly fluorescent compound fluorescein, that is measured in this method, as described previously [19]. Briefly, frozen muscle was ground using a mortar and pestle under liquid nitrogen and homogenised in 0.5% hexadecyltrimethylammonium bromide in PBS. Samples were centrifuged and supernatants diluted in PBS. Human MPO was used as the standard for the assay (Cayman Chemical). Aliquots of each experimental sample or MPO standard were pipetted into a 384 well plate, before the addition of APF working solution (20 μM APF and 20 μM hydrogen peroxide in PBS) was added. The plate was incubated at room temperature (protected from light) for 30 minutes, with fluorescence measured every minute using excitation at 485 nm and emission at 515–530 nm. The rate of change of fluorescence for each sample was compared to that of the standards and results were expressed per mg of protein, quantified using the DC protein assay (Bio-Rad).

### Quantification of protein thiol oxidation

Reduced and oxidized protein thiols were measured in quadriceps muscle using the 2 tag technique as described previously [19]. In brief, frozen tissue was crushed under liquid nitrogen, before protein was extracted with 20% trichloroacetic acid (TCA)/acetone. Protein was solubilized in 0.5% sodium dodecyl sulphate (SDS) with 0.5 M Tris at pH 7.3 (SDS buffer) and protein thiols were labeled with the fluorescent dye BODIPY FL-N-(2-aminoethyl) maleimide (FLM, Invitrogen). Following removal of the unbound dye using 100mM cysteine, oxidized thiols were reduced with tris(2-carboxyethyl)phosphine (TCEP) before the subsequent unlabeled reduced thiols were labeled with a second fluorescent dye Texas Red C2-maleimide (Texas red, Invitrogen). The sample was washed in acetone and re-suspended in SDS buffer. Samples were read using a fluorescent plate reader (Fluostar Optima) with wavelengths set at excitation 485 nm, emission 520 nm for FLM and excitation 595 nm, emission 610 nm for Texas red. A standard curve for each dye was generated using ovalbumin and results were expressed per mg of protein, quantified using the DC protein assay (Bio-Rad).

### HPLC analysis of taurine and cysteine

Taurine and cysteine levels in liver, plasma and quadriceps muscle were measured using reverse phase high performance liquid chromatography (HPLC) as previously described [30]. In brief, frozen tissues were crushed using a mortar and pestle under liquid nitrogen and homogenized in 25 times 5% TCA. Plasma samples were precipitated by addition of 10 times by volume of 5% trichloroacetic acid (TCA). After centrifugation, supernatants were removed and stored at -80°C until analysis. Analytes were separated using HPLC with fluorescent detection, with pre-column derivatisation with o-phthalaldehyde (OPA) and 2-mercaptoethanol (2ME). OPA reacts rapidly with amino acids and sulfhydryl groups to yield intensely fluorescent derivatives, and 2ME, a reducing agent, prevents the OPA reagent from oxidising. Supernatants were mixed with iodoacetamide, dissolved in 5% TCA, to a final concentration of 25 mM. An internal standard, o-phospho-dl-serine, dissolved in 5% TCA was added to a final concentration of 5 mM. Sodium borate was used to adjust the pH to 9. Samples were placed in an autosampler, which was maintained at 4°C. Samples were mixed on a sample loop with a derivitising solution containing 40 mM OPA and 160 mM 2ME in 100 mM sodium borate, pH 12, for 30 seconds before injection onto the column. Separation was achieved with a C18 column (5 μl, 4.6 x 150 mm, Phenomenex) using a Dionex Ultimate 3000 HPLC system. Mobile phase A consisted of 50 mM potassium phosphate buffer and tetrahydrofuran (97:3). Mobile phase B consisted of 90% methanol, with a gradient increase in B from 0 to 25%. Fluorescence was set at 360 nm and 455 nm for excitation and emission respectively. The protein content of liver and muscle samples were quantified by solubilising the pellet in 0.5 M sodium hydroxide, before incubation at 80°C for 15 minutes. Once fully dissolved, protein concentrations of supernatants were quantified using a Bradford protein assay (Bio-Rad).

### Protein extraction and immunoblotting

Frozen livers and quadriceps muscles were crushed using a mortar and pestle under liquid nitrogen and homogenized in 1 ml ice-cold 1% NP40, 1 mM EDTA in phosphate buffered saline (PBS), supplemented with complete EDTA free protease inhibitor tablets and PhosSTOP phosphatase inhibitor tablets (Roche), and centrifuged at 10,000 *g* for 10 min. The protein concentrations of supernatants were quantitated using the DC protein assay (Bio-Rad). Samples were resolved on 4–15% SDS-PAGE TGX Stain-Free gels (Bio-Rad) and transferred onto nitrocellulose membrane using a Trans Turbo Blot system (Bio-Rad), after imaging for total protein using the Stain-Free imaging program on the ChemiDoc MP Imaging System (Bio-Rad). Immuno-blotting was performed with rabbit polyclonal antibodies to neutrophil elastase (ab68672, Abcam), cysteine dioxygenase type 1 (ab53436, Abcam) and cysteine sulfinate decarboxylase (ab101847, Abcam) all dissolved 1:1000 in 5% bovine serum albumin (BSA). HRP-conjugated goat anti-rabbit secondary antibodies were from Thermo Fisher Scientific. Chemiluminescence signal was captured using the ChemiDoc MP Imaging System (Bio-Rad). Resultant images were quantified using ImageJ software [31]. A common sample was loaded onto each gel to normalise for detection efficiencies across membranes, and all bands were standardised to total protein for that lane [32].

### Cysteine dioxygenase activity

Cysteine dioxygenase activity was measured by quantification of cysteine sulfinate from substrate cysteine, as per [33], with some modifications. In brief, frozen livers were crushed using a mortar and pestle under liquid nitrogen and homogenized in 10 times 200 mM 2-(N-Morpholino)ethanesulfonic acid (MES) at pH 6.1. After centrifugation, supernatants were removed and aliquoted into two tubes (one labelled blank). Remaining supernatant was retained and the protein concentration quantitated using the DC protein assay (Bio-Rad). To both other tubes, a mixture containing 2.5 mM ferrous sulphate, 10 mM NAD^+^ and 25 mM hydroxylamine hydrochloride at pH 5.9 was added. To both tubes, a mixture containing 40 mM cysteine and 0.50 mM bathocuproinedisulfonic acid was added. 20% sulfosalicylic acid was immediately added to the blank tubes, and all samples were incubated for 5 minutes. 20% sulfosalicylic acid was added to the rest of the tubes, and samples were incubated on ice for 15 minutes before centrifugation to remove the precipitated protein. Supernatants were removed and stored at -80°C before analysis. Analytes were separated using HPLC with fluorescent detection, with pre-column derivitisation with OPA and 2ME as per analysis of taurine and cysteine as above. Activity of cysteine dioxygenase was determined to be the amount of cysteine sulfinate formed per minute, by subtracting the cysteine sulfinate content of the sample with cysteine substrate added minus the cysteine sulfinate content of the sample without cysteine substrate, divided by 5 (samples were incubated for 5 minutes).

### Statistics

Data were analysed using GraphPad Prism software. One-way ANOVA tests with post-hoc (LMS) comparisons were used to identify significant differences between experimental groups. Two-way repeated measures ANOVAs were used to analyse data for the force-frequency protocols. Statistical significance was accepted at p<0.05. Phenotype and force data are presented as mean ± SEM, all data points are presented ± SEM for all other measures.

## Results

### Phenotype data

Body, muscle and liver weights were measured at 6 weeks of age to identify potential detrimental effects of treatment with taurine in young growing mdx mice. While body weights were similar for C57 and untreated mdx mice, taurine treatment reduced mdx body weight by 22% (Table 1). Liver weights were similar across all groups. Quadriceps and EDL muscles were heavier in untreated mdx mice compared with C57 mice (44% and 18% respectively). Taurine treatment reduced mdx quadriceps and EDL muscle weight by ~30% (Table 1). Tibia length was similar for C57 and mdx mice (Table 1), and taurine treatment reduced mdx tibia length by 12%. EDL cross sectional area (to which force recordings were normalised) was similar for C57 and mdx mice (Table 1), however, taurine treatment reduced the mdx EDL cross sectional area by 25%. These data indicate that treating juvenile mdx mice with a high dose of taurine (16 g/kg/day) causes growth restriction.

**Table 1:** Phenotypic data of untreated C57, untreated mdx and taurine treated mdx, including body and liver and quad and EDL weight (wt), tibia length and EDL cross sectional area (CSA).

	Untreated C57	Untreated mdx	Taurine mdx
Body wt (g)	19.95 ± 0.52	20.56 ± 0.65	16.06 ± 0.59^$^
Liver wt (g)	1.00 ± 0.05	1.05 ± 0.09	0.93 ± 0.06
Quad wt (mg)	95.5 ± 4.5	137.9 ± 4.5*	99.1 ± 5.2^$^
EDL wt (mg)	5.6 ± 0.4	6.6 ± 0.3*	4.6 ± 0.3^$^^#^
Tibia length (mm)	16.8 ± 0.16	17.0 ± 0.3	15.2 ± 0.3^$^^#^
EDL CSA (mm^2^)	1.35 ± 0.07	1.34 ± 0.06	1.01 ± 0.05^$^^#^

Symbols for significant differences (p<0.05) are-

* = between untreated mdx and C57

$ = between untreated mdx and taurine treated mdx and

# between taurine treated mdx and C57. Data are presented as mean ± SEM and n = C57 (9), mdx (10) and taurine treated (8).

### Grip strength and *ex vivo* muscle strength

**Fig 1:**
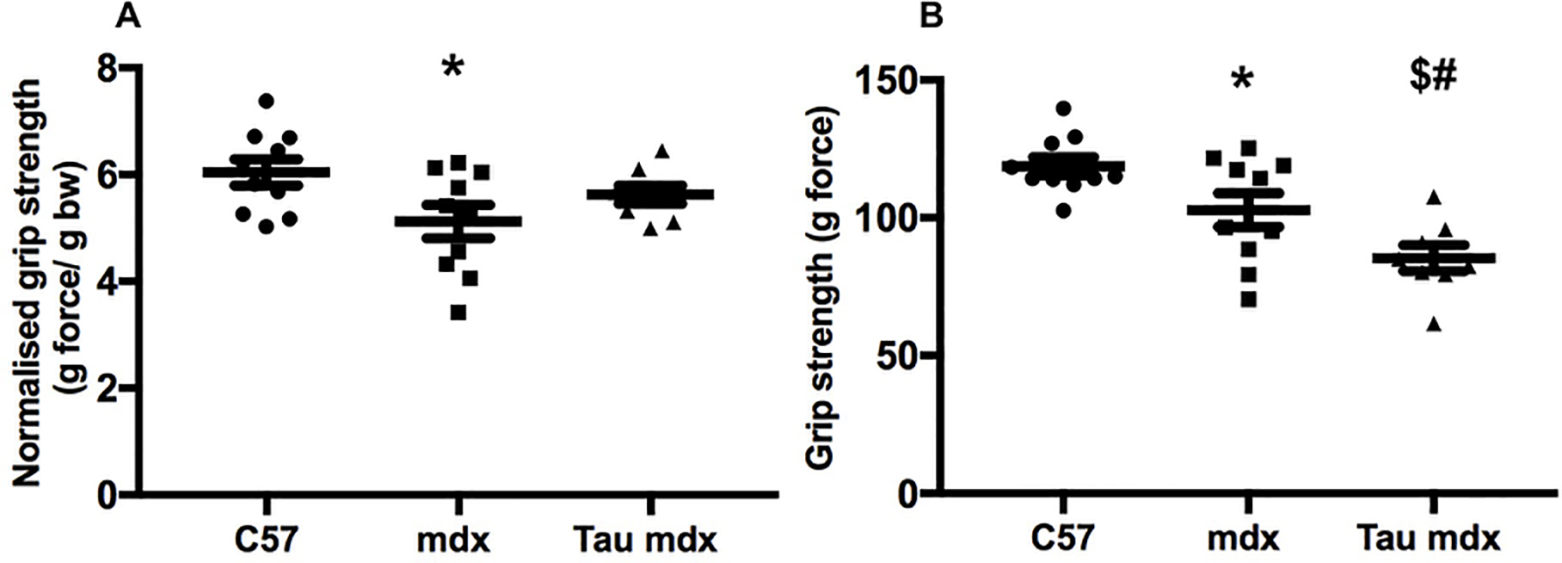
Fore limb grip strength of untreated C57, untreated mdx and taurine treated mdx mice. Data are presented as both force normalised to body weight (A) and total force (B). Symbols for significant differences (p<0.05) are- * = between untreated mdx and C57, $ = between untreated mdx and taurine treated mdx and # between taurine treated mdx and C57. Data are presented as mean ± SEM and n = C57 (9), mdx (10) and taurine treated (8).

The grip strength test is a non-invasive *in vivo* measure of mouse limb strength. Mdx mice had weaker (15%) normalised (to body weight) grip strength (Fig 1A) and total grip strength (Fig 1B), compared with C57 mice. Although taurine treatment of mdx mice had no effect on normalised grip strength (Fig 1A), there was a 15% reduction in total grip strength (Fig 1B).

**Fig 2:**
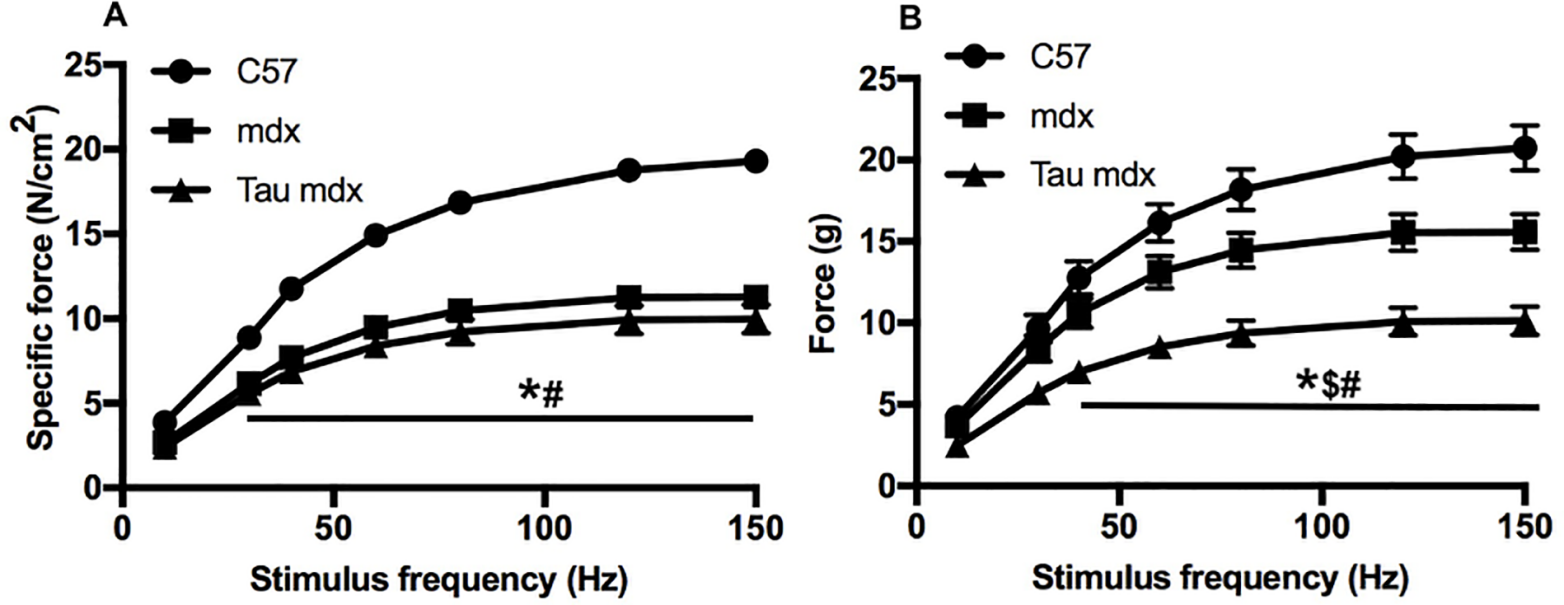
Force frequency curves of untreated C57, untreated mdx and taurine treated mdx EDL muscles. Isometric force produced at frequencies from 5–150 Hz was plotted for both specific force (A) and total force (B). Symbols for significant differences (p<0.05) are- * = between untreated mdx and C57, $ = between untreated mdx and taurine treated mdx and # between taurine treated mdx and C57. Data are presented as mean ± SEM and n = C57 (7), mdx (9) and taurine treated (7).

Mdx EDL muscle produced lower specific tetanic forces *ex vivo* at stimulation frequencies of 30 Hz and above (Fig 2A), and lower total tetanic forces at stimulation frequencies of 40 Hz and above (Fig 2B), compared with C57 EDL muscle. While taurine treatment had no effect on specific tetanic force of mdx EDLs (Fig 2A), there was a reduction in total tetanic force at stimulation frequencies of 40 Hz and above (Fig 2B). These data show that the high taurine dose did not improve either *in vivo* or *ex vivo* muscle strength.

### Inflammation and protein thiol oxidation

**Fig 3:**
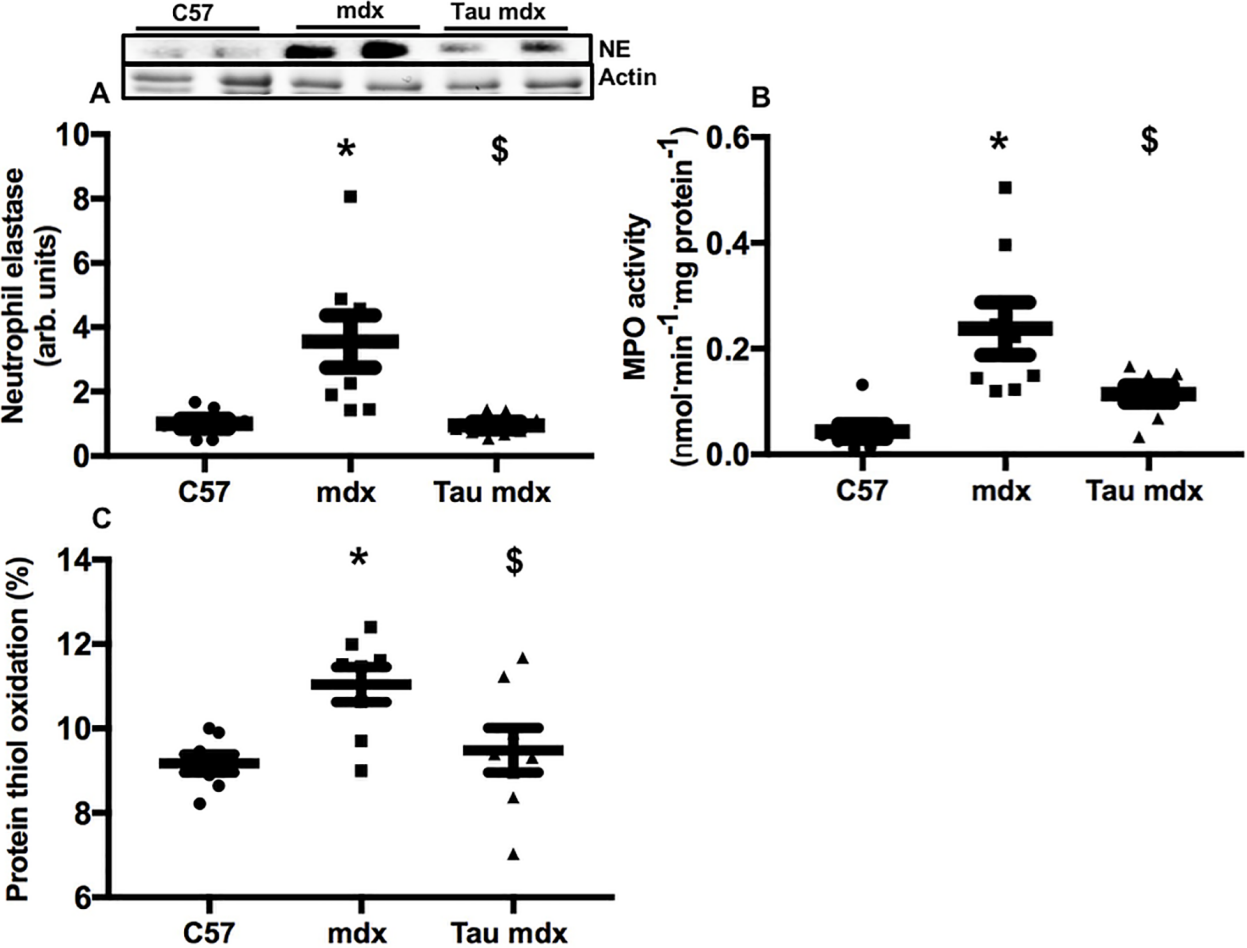
**Quadriceps muscle content of neutrophil elastase (A) and MPO (B) and percentage of protein thiol oxidation (C) in untreated C57, untreated mdx and taurine treated mdx mice.** Symbols for significant differences (p<0.05) are- * = between untreated mdx and C57, $ = between untreated mdx and taurine treated mdx. Data are presented as mean ± SEM and n = C57 (8), mdx (8) and taurine treated (8). A representative blot for neutrophil elastase is shown, protein was standardised to total protein using stain-free gels. From the same stain-free gel image, the loading of actin is shown.

Neutrophil content was 250% higher in mdx muscle compared with C57 muscle (Fig 3A), and taurine treatment reduced, by 70%, neutrophil content of mdx muscle back to C57 levels. MPO content was 440% higher in mdx muscle compared with C57 muscle (Fig 3B), and taurine treatment reduced, by 50%, MPO content of mdx muscle. Protein thiol oxidation was 20% higher in mdx muscle compared with C57 muscle (Fig 3C), and taurine treatment reduced, by 20%, protein thiol oxidation in mdx muscle, back to levels in C57 mice. Overall, the high dose taurine had striking benefits on these parameters in the dystrophic mdx muscles.

### Taurine and cysteine content of liver, plasma and muscle

**Fig 4:**
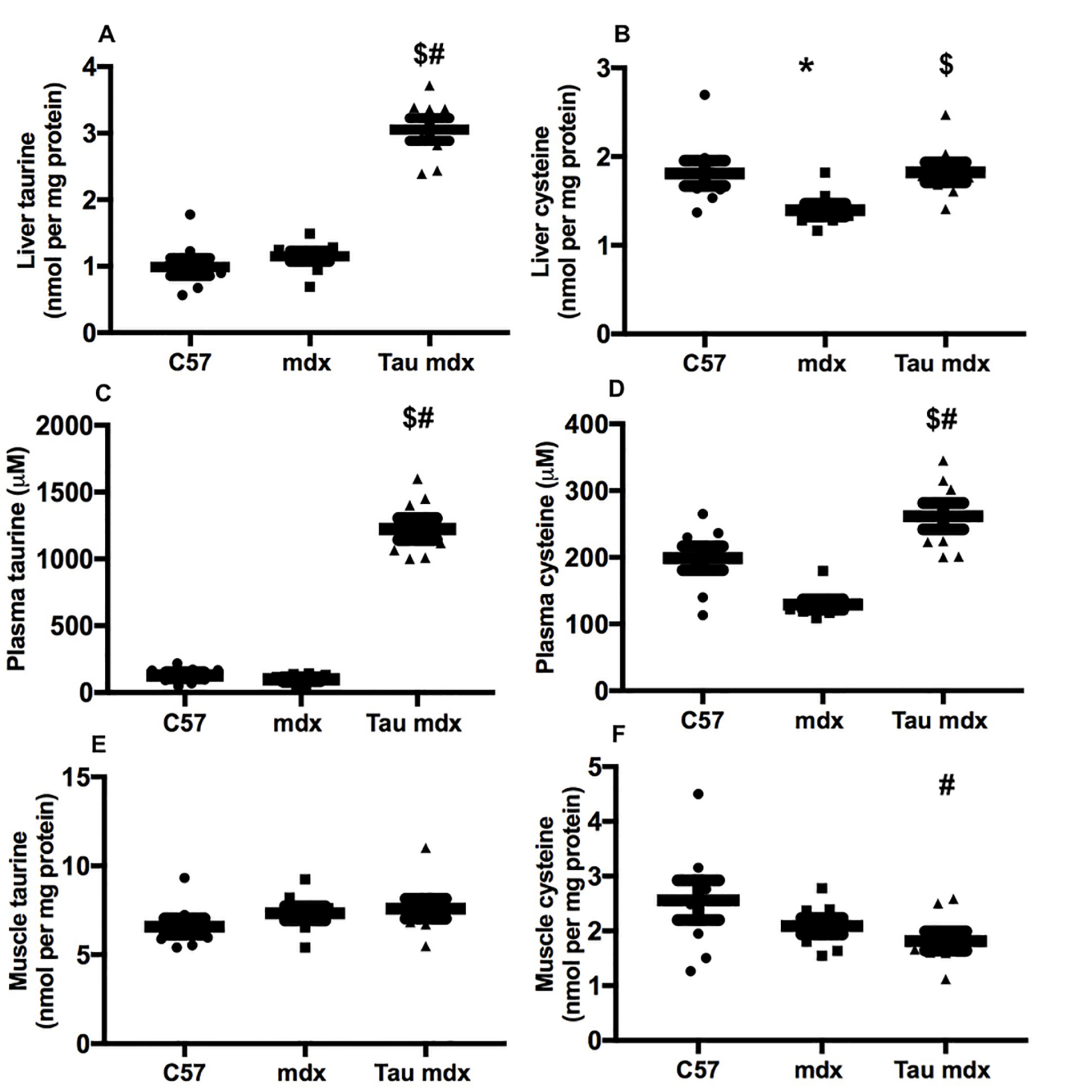
**Liver content of taurine (A) and cysteine (B), plasma content of taurine (C) and cysteine (D) and quadriceps muscle content of taurine (D) and cysteine (E) in untreated C57, untreated mdx and taurine treated mdx mice.** Symbols for significant differences (p<0.05) are- * = between untreated mdx and C57, $ = between untreated mdx and taurine treated mdx and # between taurine treated mdx and C57. Data are presented as mean ± SEM and n = C57 (8), mdx (8) and taurine treated (8).

Taurine content was similar for C57 and mdx liver (Fig 4A), and taurine treatment led to a 200% increase in taurine content of mdx liver. Taurine content was similar for C57 and mdx plasma (Fig 4C), and taurine treatment increased taurine content of mdx plasma by 1100%. Muscle taurine content was similar for all groups (Fig 4E). Cysteine content was 20% lower in mdx liver compared with C57 (Fig 4B), and taurine treatment increased, by 30%, cysteine content of mdx liver, back to C57 levels. Cysteine content was 35% lower in mdx plasma compared with C57 (Fig 4D), and taurine treatment increased cysteine content of mdx liver by 100%, with cysteine content of taurine treated mdx plasma being also 33% higher than C57 plasma. Muscle cysteine content was similar for C57 and mdx groups; however cysteine content of taurine treated mdx muscle was 30% lower that C57 muscle (Fig 4F).

These data indicate that delivery of the high dose of taurine (16 g/kg/day) in the drinking water to mdx mice is highly effective at increasing levels of taurine in the liver and plasma; however, this high dose taurine causes a (potentially toxic) build-up of cysteine.

### Liver cysteine dioxygenase and cysteine sulfinate decarboxylase

**Fig 5:**
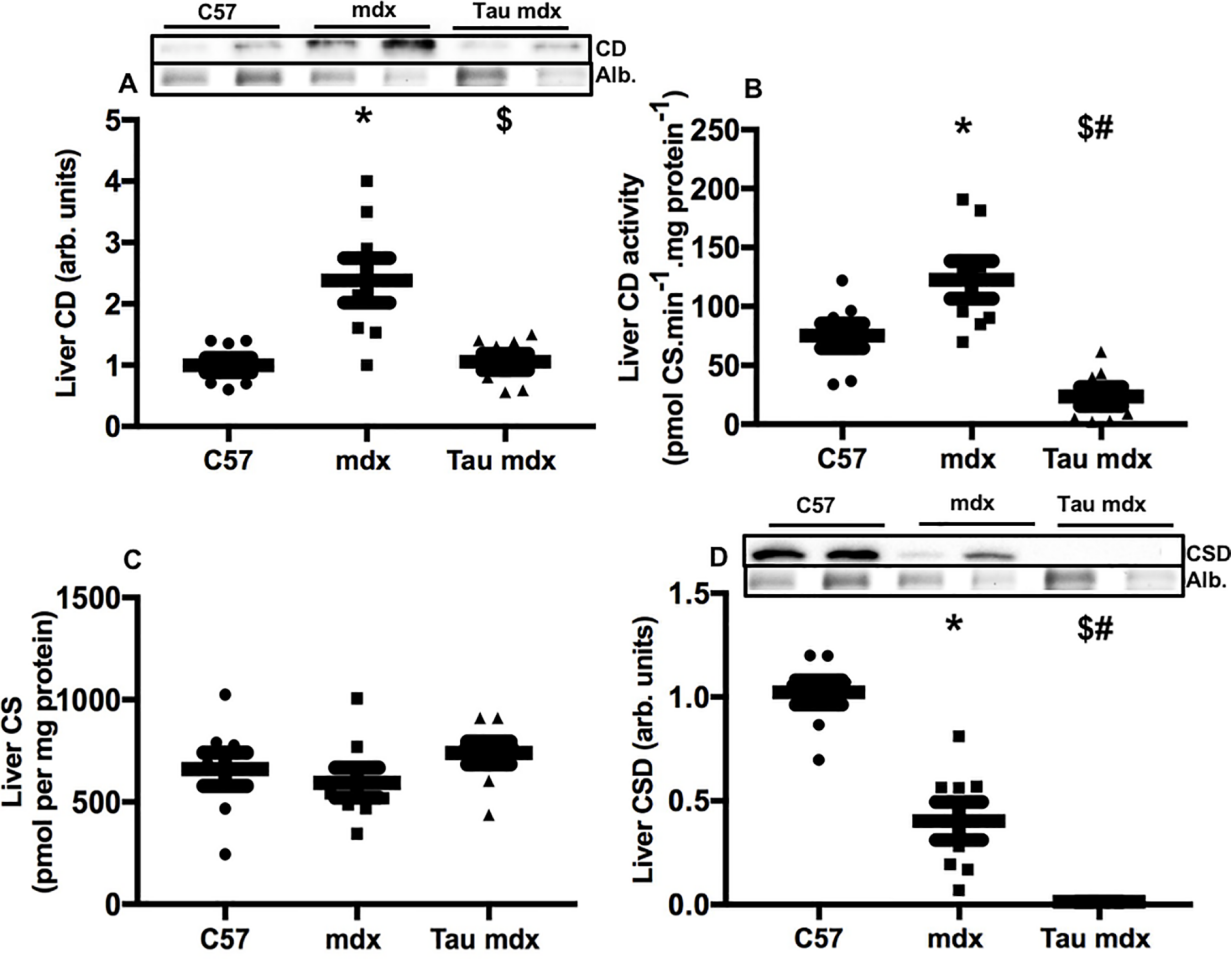
**Liver content (A) and activity (B) of cysteine dioxygenase, content of cysteine sulfinate (C) and cysteine sulfinate decarboxylase (D) in untreated C57, untreated mdx and taurine treated mdx mice.** CD = cysteine dioxygeasne, CS = cysteine sulfinate, CSD = cysteine sulfinate decarboxylase. Symbols for significant differences (p<0.05) are- * = between untreated mdx and C57, $ = between untreated mdx and taurine treated mdx and # between taurine treated mdx and C57. Data are presented as mean ± SEM and n = C57 (8), mdx (8) and taurine treated (8). Representative blots are shown, proteins were standardised to total protein using stain-free gels. From the same stain-free gel image, the loading of albumin is shown.

Immunoblotting quantification of protein levels of enzymes related to cysteine metabolism showed that cysteine dioxygenase protein was 140% higher in livers of untreated mdx mice compared with C57 (Fig 5A), and taurine treatment reduced, by 60%, cysteine dioxygenase content of mdx liver back to levels of C57 liver. Measurement of enzymatic activity showed that cysteine dioxygenase activity was 60% higher in mdx liver compared with C57 (Fig 5B), and taurine treatment dramatically decreased, by 80%, the cysteine dioxygenase activity of mdx liver, to well below the activity of C57 livers. Cysteine sulfinate (the metabolite intermediary between cysteine and taurine) content was similar for all groups (Fig 5C). Cysteine sulfinate decarboxylase was 60% lower in mdx liver compared to C57 liver (Fig 5D), and taurine treatment reduced cysteine sulfinate decarboxylase content of mdx liver by 97%.

These data show that treating juvenile mdx mice with a high dose of taurine likely causes a down-regulation of taurine synthesis in mdx liver, leading to a build-up of cysteine in mdx liver and plasma.

## Discussion

The key novel observation in this study is that delivery of high dose taurine in drinking water, estimated to be about 16 g/kg/day, caused growth deficits in young mdx mice. We propose that the cause of the growth deficit is an indirect consequence of an increase in cysteine levels resulting from an inhibition of the taurine synthesis pathway (which disposes of excess cysteine) in mdx mice. Nevertheless, consistent with previous work, there was good evidence that taurine decreased inflammation and protein oxidation in dystrophic tissue.

We and others have shown that taurine treatment of mdx mice (with doses ranging from 1 to 16 g/kg/day) decreases dystropathology, with an improvement in muscle strength and a decrease in myofibre necrosis, inflammation and oxidative stress [15–21]. Likewise, we show in the current study using young juvenile mdx mice that a high taurine dose of 16 g/kg/day (from 7 days until 6 weeks of age) decreased neutrophil and MPO content of mdx muscle, as well as protein thiol oxidation, supporting our hypothesis that taurine exerts strong anti-inflammatory and antioxidants benefits in dystrophic muscle. We propose a likely mechanism involves scavenging of HOCl by taurine, and subsequent formation of the anti-inflammatory molecule taurine chloramine [22, 24, 25].

The high dose taurine in the present study resulted in a decrease in total *in vivo* and *ex vivo* force. However this dose did not affect *in vivo* and *ex vivo* muscle strength when normalised to body weight (grip strength) or to cross sectional area calculated from muscle weight (specific force). This indicates that taurine was not detrimental to the inherent contractile properties of muscle(s). Instead, the loss of force can be attributed to smaller body and muscle weights in taurine treated young mdx mice, relative to age-matched untreated mdx mice.

The decrease in body weight and muscle size was also associated with a decrease in tibia length of taurine treated mdx mice, indicating that the high dose taurine caused growth restriction in these young mdx mice. Whilst studies have shown that dietary taurine restriction can lead to growth restriction [34, 35], evidence that excess taurine can also cause growth restriction is limited in the literature. One study showed that treating newly weaned pigs for 4 weeks with 3% taurine (in food) significantly reduced the weight gain to feed ratio, suggesting some effect of taurine treatment on growth rate, although this did not affect final body weight [36]. Additionally, feeding juvenile sablefish with a range of taurine concentrations (from 0.25% to 6% in food) resulted in growth restriction at low and high concentrations of taurine, with optimal weight gain occurring between 0.4% and 4.2% dietary taurine [37].

Our data indicate that a high dose of taurine may not be an effective or safe treatment for very young growing mdx mice. In contrast, De Luca and colleagues did not observe any deleterious effects of taurine when treating mdx mice aged 4 weeks (until 8 weeks) with an equivalently high dose of taurine (up to 400 mg per mouse per day, which is comparable with our dose of 16 g/kg/day). Their dose was also highly effective at improving strength of exercised mdx muscles, and was more beneficial than treatment with prednisolone, the current gold standard treatment of DMD boys [16]. There are a number of possible explanations for the discrepancy between our study and that of De Luca. In the De Luca study, mice underwent chronic treadmill exercise, which may have had effects on metabolism or protein turnover that may have ameliorated any toxic effects of high taurine. Additionally, taurine treatment of young mdx mice in the De Luca study began at 4 weeks, while in the current study mice had access to taurine enriched water from 7 days of age. The growth phase of mice is relatively very short, with rapid growth up until 6 weeks before a marked decline of growth until almost ceases by 10–12 weeks [38]. By treating mice from 1–6 weeks of age, we captured a greater portion of their period of maximal growth, whereas mice aged 4–8 weeks in the De Luca study missed the important earlier intense growth phase, with associated age-related changes in metabolism. Regardless of the speculative causes for the discrepancy, results from the current study indicate that taurine treatment regimes can have unforeseen side effects. The usefulness of taurine as a treatment for dystrophy would benefit from further research into the circumstances (e.g. dosing, exercise) in which the side-effects are manifested.

We propose that the detrimental effects of taurine on growth are an indirect effect caused by increased cysteine levels in the liver and plasma. Excess cysteine is considered toxic (reviewed in [39]) and excess dietary cysteine is known to reduce growth rates [40]. We have previously shown that treating mdx mice with the cysteine precursor OTC from weaning to 6 weeks led to growth restriction [19] similar to that observed in this current study. In the present study the high dose of taurine increased cysteine levels in mdx liver and plasma and it seems likely that this resulted from a down-regulation of taurine synthesis in the liver, which functions to dispose of excess cysteine [41].

Cysteine is catabolised by the enzyme cysteine dioxygenase to cysteine sulfinate, which is decarboxylated by the enzyme cysteine sulfinate decarboxylase to form hypotaurine, which is then oxidised to taurine [42]. We found that the high dose of taurine decreased cysteine dioxygenase content and activity, and dramatically decreased cysteine sulfinate decarboxylase content in mdx liver. The activity and levels of these enzymes are highly sensitive to cysteine liver content and dietary intake of cysteine, as the body pool of cysteine requires tight regulation. While cysteine is toxic at high levels, adequate levels are required for protein and glutathione synthesis [39].

Our observation that taurine exerts a strong feedback effect on this pathway appears to be novel. Although a high protein diet has also been shown to affect activity and levels of these enzymes, this response was suggested to be wholly dependent on the sulfur containing amino acids cysteine and methionine within the protein diet [43–46]. One relevant study showed that cysteine sulfinate decarboxylase was subject to end-product feedback inhibition, since dietary taurine supplementation in healthy rats caused a significant decline in activity [47]. We were not able to locate any studies that suggested that taurine could cause toxicity as a result of excess cysteine. Taurine therapy in adult humans has been reported to be safe and non-toxic, with no adverse effects reported (with the exception of minor gastrointestinal issues) [48]. Our observations are in mdx mice only, and we have previously shown that taurine synthesis is dysregulated in mdx mice [26] and there appear to be different metabolic demands in dystrophic mdx mice [49]. Such differences may render mdx mice more sensitive to cysteine toxicity (caused by a taurine treatment), compared with other mouse strains; this is supported by research [21] showing that while taurine treatment (3% in drinking water) decreased weight of adult mdx mice, C57 mice were not affected. Further research is required to establish if high doses of taurine are unsafe in other animal models and humans.

We and others have provided strong data from pre-clinical studies to support the use of taurine as a highly promising and inexpensive therapeutic nutritional intervention for DMD. The issue of growth restriction with high taurine dosage in very young dystrophic animals adds a cautionary note. We propose that this concern can be addressed by carefully monitoring levels of metabolites of taurine and cysteine in the blood and urine of growing animal models and DMD boys. As a consequence, further research is required to identify the dosing range of taurine that maximises the protective benefits of taurine while minimising the likelihood of growth restriction, before considering clinical translation to young DMD boys.

## Supporting information

S1 Table Mean values for male and female mice individually for all indices. Symbol (*) denotes significant differences (p<0.05) between male and female mice in that treatment group.
(DOCX)
