# Supplementary material for: Beneficial effects of high dose taurine treatment in juvenile dystrophic mdx mice are offset by growth restriction
Source: PLoS One. 2017 Nov 2;12(11):e0187317. doi: 10.1371/journal.pone.0187317 (PMC5667875; doi:10.1371/journal.pone.0187317)
Supplement: S1 Table — Symbol (*) denotes significant differences (p<0.05) between male and female mice in that treatment group. (DOCX) [file pone.0187317.s001.docx]

|  | C57  Male Female | | Mdx  Male Female | | Taurine mdx  Male Female | |
| --- | --- | --- | --- | --- | --- | --- |
| Body wt | 20.82±1 | 19.26±0.2 | 21.91±1.2 | 19.98±0.7 | 16.41±1 | 15.85±0.9 |
| Liver wt | 1.17±0.06 | 0.76±0.03* | 1.07±0.15 | 1.08±0.13 | 0.82±0.02 | 1.03±0.1 |
| Quad wt | 99.67±6.4 | 87.27±4.7 | 150.77±3.4 | 131.42±5.2* | 102.7±6.6 | 95.43±8.4 |
| EDL wt | 6.38±0.5 | 4.96±0.4 | 7.1±1 | 6.45±0.2 | 5.07±0.7 | 4.26±0.3 |
| Tibia length | 17.04±0.1 | 16.38±0.2 | 16.75±0.5 | 17.09±0.3 | 15.04±1.1 | 15.38±0.3 |
| EDL CSA | 1.30±0.1 | 1.40±0.1 | 1.50±0.2 | 1.27±0.04 | 1.13±0.1 | 0.93±0.04 |
| Norm. grip | 5.90±0.5 | 6.13±0.3 | 4.09±0.4 | 5.55±0.3 | 5.49±0.3 | 5.70±0.3 |
| Grip strength | 121.67±4 | 116.67±5.5 | 87.72±4.6 | 109.24±7.3 | 80.67±1.5 | 88.2±7.6 |
| Neut. elast. | 0.98±0.3 | 1.01±0.2 | 2.52±0.7 | 4.17±1.2 | 0.91±0.2 | 0.98±0.2 |
| MPO | 0.42±0.2 | 0.17±0.05 | 0.83±0.05 | 1.79±0.4 | 0.74±0.06 | 0.63±0.2 |
| Thiol ox. | 8.80±0.3 | 9.40±0.3 | 11.53±0.04 | 10.48±0.9 | 8.90±0.9 | 10.07±0.6 |
| Liv. taurine | 1.30±0.3 | 0.80±0.1 | 1.11±0.2 | 1.17±0.06 | 3.12±0.3 | 2.99±0.2 |
| Liv. cysteine | 2.05±0.3 | 1.67±0.1 | 1.48±0.2 | 1.35±0.1 | 1.91±0.2 | 1.74±0.1 |
| Plas. taurine | 113.50±29.5 | 137.95±29.8 | 62.37±6.2 | 122.79±8.7 | 1167±83.3 | 1332±189 |
| Plas. cysteine | 228.26±18.7 | 181.11±24.2 | 124.20±3.6 | 132.14±12.5 | 232.95±17.6 | 323.50±15.4 |
| Mus. taurine | 7.32±1.1 | 6.14±0.3 | 7.93±0.7 | 7±0.5 | 6.96±0.5 | 8.21±1 |
| Mus. cysteine | 1.98±0.6 | 2.91±0.4 | 2.3±0.1 | 1.96±0.2 | 1.55±0.2 | 2.08±0.3 |
| Liv. CD | 1.17±0.2 | 0.9±0.1 | 2.47±0.7 | 2.33±0.5 | 0.86±0.2 | 1.25±0.2 |
| Liv. CD act. | 64.17±28.9 | 81.68±5 | 121.97±34.4 | 122.89±19 | 24.38±11 | 23.05±11.8 |
| Liv. CS | 719.35±163.7 | 624.81±100 | 623.26±198.3 | 576.94±54.3 | 764.24±88.9 | 725.20±77.8 |
| Liv, CSD | 0.99±0.06 | 1.04±0.1 | 0.47±0.1 | 0.36±0.1 | 0.01±0.0002 | 0.02±0.002 |

**S1 Table. Mean values for male and female mice individually for all indices.** Symbol (*) denotes significant differences (p<0.05) between male and female mice in that treatment group.
